# Prevalence of and factors associated with scrub typhus exposure among the hill tribe population living in high incidence areas in Thailand: a cross-sectional study

**DOI:** 10.1186/s12889-023-17313-z

**Published:** 2023-12-01

**Authors:** Nidanuch Tasak, Tawatchai Apidechkul, Andrew C. K. Law, Mohammad Yazid Abdad, Peeradone Srichan, Carlo Perrone, Ampai Tanganuchitcharnchai, Jantana Wongsantichon, Stuart D. Blacksell

**Affiliations:** 1 School of Health Science, Mae Fah Luang University, Chiang Rai, Thailand; 2 Mahidol Oxford Tropical Medicine Research Unit, Bangkok, Thailand; 3 Center of Excellence, The Hill Tribe Health Research, Mae Fah Luang University, Chiang Rai, Thailand; 4 Department of Psychiatry, Royal College of Surgeons in Ireland & University College Dublin, (Malaysia Campus), Pulau Pinang, Malaysia; 5 Centre for Tropical Medicine and Global Health, University of Oxford, Oxford, UK

**Keywords:** Hill tribe, Scrub typhus, Prevalence, Factor associated

## Abstract

**Background:**

Scrub typhus is a significant tropical disease, occurring in rural settings and therefore usually afflicting remote agricultural populations who have lower socioeconomic status and limited access to medical care. A large proportion of the hill tribe people in Thailand are financially poor, have limited education, and do not have adequate health care access. This study aimed to estimate the prevalence of and determine factors associated with scrub typhus exposure among the hill tribe population living in high-incidence areas in northern Thailand.

**Methods:**

A cross-sectional study design was used to gather information from hill tribe people aged 18 years and over living in ten hill tribe villages in Mae Fah Luang, Chiang Rai Province, Thailand. Participants who met the inclusion criteria were invited to participate in the study. A validated questionnaire was used as the research instrument, and 5 mL blood samples were taken. *Orientia tsutsugamushi* IgM and IgG antibodies were detected by enzyme-linked immunosorbent assay (ELISA) and then confirmed by immunofluorescence assay (IFA). Logistic regression was used to detect associations between variables at a significance level of α = 0.05.

**Results:**

A total of 485 hill tribe people participated in the study; 57.1% were female, 29.9% were over 60 years of age, 46.4% were from the Akha tribe, and 74.2% had never attended school. The overall prevalence of scrub typhus exposure was 48.0%. In the multivariate model, five variables were found to be associated with scrub typhus exposure. Participants aged over 60 years had a 4.31-fold increased risk (95% CI = 1.73–10.72) of scrub typhus exposure compared to those who were younger than 30 years. Those who were illiterate had a 3.46-fold increased risk (95% CI = 1.93–6.21) of scrub typhus exposure than those who had at least a primary education level. Participants from the Akha tribe had a 2.20-fold increased risk (95% CI = 1.31–3.72) of scrub typhus exposure than those from the Lahu tribe. Subjects who had a history of cutting grass had a 1.85-fold increased risk (95% CI = 1.20–2.84) of scrub typhus exposure. Those who never wore gloves for farming had a 2.12-fold increased risk (95% CI = 1.28–3.49) of scrub typhus exposure than those who wore gloves daily.

**Conclusions:**

There is a high prevalence of scrub typhus exposure among the hill tribe in Thailand. Effective public health interventions to promote scrub typhus awareness and prevention are urgently needed in these populations.

## Introduction

Scrub typhus is caused by bacteria from the genus *Orientia*, namely *Orientia tsutsugamushi, Candidatus* Orientia chuto*,* and *Candidatus* Orientia chiloensis [1, 2]. It is considered one of the most significant tropical diseases globally [3]. The annual global number of scrub typhus infections is approximately one million, and individuals living in rural areas are particularly vulnerable to the disease [4], particularly those living in the Asia–Pacific region [4, 5]. The infection can lead to several serious complications, especially in the lungs, kidneys, liver, and brain, which can cause multiorgan failure and death [3]. The case-fatality rate can be up to 70%, depending on the patient’s age, geographical area, severity stage, and complications [6]. Misdiagnosis and treatment delays due to inadequate resources are alarming and often result in poor outcomes [7, 8]. In addition, scrub typhus is a vector-borne disease that is transmitted to humans through the bite of infected chiggers. Chiggers normally feed on small mammals, especially wild rodents [9]. The infection is highly dependent on the interaction of humans with the environment [9]. Some activities, such as sitting directly on the ground, working long hours in fields, working without protective clothing, infrequent washing of clothes and skin, dry field farming, and working with livestock, have been associated with scrub typhus infection [9–11]. Environmental factors such as having water or rodents in the domestic or peri-domestic environment are also significant risk factors for scrub typhus infection [9–12].

Thailand is located in a tropical zone, and the northern region has been reported as the highest endemic area of the disease, with an average incidence rate of 9.40 cases per 100,000 people [13]. Chiang Rai Province, which borders Myanmar in the west and Laos in the east, was the province with the highest number of cases of the disease in Thailand reported between 2003 and 2018 [14]. There are 18 districts in Chiang Rai Province, with more than 1.2 million people performing agricultural practices [15], and 15–20% are hill tribe people who have specific cultures, farming practices, and clothing. Hill tribe people have a poorer socioeconomic status and live far from cities, which leads to poor access to health care services [16].

Mae Fah Luang District is the preferred living area of hill tribe people in Chiang Rai Province and shares a long border with Myanmar in the west. Approximately 86,000 people live in 88 villages in this area. Hill tribe people have been officially identified as a major underserved population by the Thai Ministry of Public Health in terms of access to health care services [16]. Basically, there are six main hill tribe groups, Akha, Lahu, Yao, Hmong, Karen, and Lisu, that have specific cultures, beliefs and lifestyles. All these populations are working in agricultural sectors to which they are easily exposed to the environment. Several barriers to accessing medical care have been clearly identified for this population: language, legal rights, distance from health care institutes, transportation difficulties, and stigmatization by health care providers [17, 18]. Thus, the study aimed to estimate the prevalence of and determine the factors associated with scrub typhus exposure among the tribe population living in villages with a high incidence of the disease in Mae Fah Luang District, Chiang Rai Province, Thailand.

## Methods

### Study design and study population

A community-based cross-sectional study was conducted in ten (10) high-incidence scrub typhus villages with cases reported between 2008 and 2017 in Mae Fah Luang District, Chiang Rai Province, Thailand [13]. The Akha, Lahu, Hmong, Yao and Lisu tribes were the study populations. Data and blood samples were collected during the period from November 1^st^, 2022, to November 30^th^, 2022. In 2021, 14,972 hill tribe people lived in the study villages [19].

The inclusion criteria for subject recruitment included a) being a hill tribe member according to verbal confirmation; b) aged 18 years or above; c) working as a farmer or in a related occupation; d) having lived in the study areas for at least 6 months at the time of survey; and e) being capable of providing informed consent. Those who could not effectively communicate to provide essential information regarding the study protocol were excluded from the study.

### Study sample and sample size calculation

The sample size was calculated based on the standard formula for a cross-sectional study [20]: *n* = [Z^2^_α/2_ P (1-P)]/e^2^, where n = the sample size needed, Z = the value from the standard normal distribution corresponding to the desired confidence level (Z = 1.96 for 95% CI), P = the expected true proportion, and e = the desired precision. Under the assumption of *P* = 0.28 [21] and e = 0.04, a sample size of 485 was calculated.

### Research instruments and their development

Participants were asked to fill out a questionnaire. The questionnaire was developed according to a literature review and discussions with field experts and was divided into three parts. Part I consisted of ten (10) questions to collect general demographic information. In Part II, 29 questions were used to collect data on behaviors related to scrub typhus exposure, such as outdoor activities, protective behaviors during and after work, animal exposures, environmental exposures, and history of scrub typhus exposure. Part III included ten (10) questions to collect information about scrub typhus prevention and control. A five (5)-mL blood sample was collected for serological analysis.

The questions were validated by using item-objective congruence (IOC) by three experts in the field: a physician and two epidemiologists who were familiar with scrub typhus. Subsequently, a pilot test was conducted among 10 hill tribe people who had characteristics similar to those of the study population in Mae Chan District, Chiang Rai Province, and Cronbach’s alpha was 0.71.

### Steps of data collection

After receiving approval from the Chiang Rai Provincial Public Health Office Ethics Committee, the Mae Fah Luang District government officer and health care providers were contacted to obtain their approval for access to the villagers. Afterward, the village headmen and village health volunteers were contacted and were provided with details regarding the study. People who met the inclusion criteria were informed and contacted seven (7) days prior to data collection.

On the date of data collection, all participants were asked to provide informed consent, after which blood collection was performed by a licensed health professional. The questionnaire was completed by interview. For those who could not understand Thai, village health volunteers who were fluent in both Thai and the local languages were asked to assist in translating the information clearly before the questions were completed. The whole process lasted approximately 30 min for each participant.

### Laboratory methods

The laboratory analysis was performed at the Mahidol-Oxford Tropical Medicine Research Unit (MORU), Bangkok, Thailand. In brief, samples were screened for *Orientia tsutsugamushi* IgM and IgG positivity (defined as an optical density of ≥ 0.50) by ELISA [22]. The MORU in house STG IgM and IgG ELISA uses specific antigens of *Orientia tsutsugamushi* Karp, Kato, Gilliam, and TA716 strains to detect scrub typhus IgM and IgG antibodies, based on the method described in the study Phanichkrivalkosil et al. [23] Whole-cell antigen lysates of Karp, Kato, and Gilliam reference strains of *Orientia tsutsugamushi* and mock-infected cell lysate produced at the Viral and Rickettsial Diseases Department of the NMRC, Silver Spring, Maryland, USA, and TA716 a local strain from Thailand produced at MORU were used [23, 24]. A confirmatory IFA is considered the gold standard to detect *Orientia tsutsugamushi* antibodies, with results reported by titer using a fluorescence microscope. Forty-well *Orientia tsutsugamushi* antigen slides were obtained from Scientific Device Laboratory, USA. Samples with a titer of 1:100 or higher were considered positive [24–26]. Scrub typhus exposure in this study was considered by reactivity to either *Orientia tsutsugamushi* IgG or IgM antibodies with the IFA method (titer of 1:100 or above).

### Measures

BMI was categorized using Asian-specific cutoffs provided by the World Health Organization: underweight: a BMI < 18.5 kg/m2; normal weight: a BMI of 18.5–22.9 kg/m2; overweight: a BMI of 23–24.9 kg/m2; and obese: a BMI ≥ 25 kg/m2 [27]. Knowledge was categorized using Bloom’s cutoff point as high knowledge if the score was between 80 and 100%, moderate knowledge if the score was between 60 and 79%, and poor knowledge if the score was less than 60% [28].

### Statistical analysis

Data were double-entered into an Excel sheet and checked for errors before being transferred into SPSS program version 20.0 (SPSS, Chicago, IL) for analysis. Continuous and categorical data were described properly to present the general characteristics of the participants and their behaviors, including scrub typhus exposure prevention behaviors. The chi-squared test was used to compare outdoor activities and behaviors related to scrub typhus exposure by tribe. Logistic regression was used to identify the factors associated with scrub typhus exposure at a significance level of α = 0.05 in both univariate and multivariate analyses. The Cox-Snell R^2^ and Nagelkerke R^2^ were used for determining the fitness of the model before interpreting the final model.

## Results

A total of 485 participants were recruited for the study. The majority were female (57.1%), aged more than 60 years (29.9%), members of the Akha tribe (46.4%), and Christian (55.9%). The majority were married (73.2%), illiterate (74.2%), had an annual income of less than 30,000 baht ($873.45) per year (41.4%), and held Thai identification cards (79.2%). Regarding knowledge about scrub typhus prevention and control, 52.8% demonstrated poor levels (Table 1).

**Table 1:** General characteristics of participants

Characteristic	n	%
**Total**	**485**	**100**
**Sex**		
Male	208	42.9
Female	277	57.1
**Age** (years)		
18–29	53	10.9
30–39	89	18.4
40–49	90	18.6
50–59	108	22.3
≥60	145	29.9
*Min* = *18, Max* = *89, Mean* = *50.20, SD* = *16.42*		
**Tribe**		
Akha	225	46.4
Lahu	123	25.4
Hmong	42	8.7
Lisu	45	9.3
Yao	50	10.3
**Marital status**		
Single	50	10.3
Married	355	73.2
Widowed/Divorce	80	16.5
**Religion**		
Buddhism	214	44.1
Christian	271	55.9
**Education**		
None	360	74.2
Primary school	55	11.3
Secondary school	58	12.0
Vocational	11	2.3
Bachelor’s degree or above	1	0.2
**Annual household income** (baht)		
< 30,000	201	41.4
30,001–50000	150	30.9
50,001–100000	104	21.4
> 100,001	30	6.2
**Having Thai identification card**		
Yes	384	79.2
No	101	20.8
**Having rights to access to health care service free of charge**		
Yes	354	73.0
No	77	15.9
Others	54	11.1
**Body mass index (BMI)**		
Underweight	26	5.4
Normal weight	157	32.4
Overweight	104	21.4
Obese	198	40.8
**Knowledge regarding scrub typhus prevention and control**		
Poor	256	52.8
Moderate	142	29.3
High	87	17.9
**Experiencing scrub typhus infection**		
Unexplained insect bite	151	31.1
Unexplained localized rash	193	39.8
Having eschar	40	8.2

Among the 485 participants, 283 (58.35%) tested positive for ELISA IgG, while 92 (18.97%) were positive for ELISA IgM. Upon confirmation, 227 (46.8%) showed IFA IgG positivity, which indicated a history of scrub typhus exposure, and 10 (2.1%) were positive for IFA IgM antibodies, which indicated recent infection. Four participants (1.72%) showed positivity for both IFA IgG and IgM antibodies. The IFA IgG titers ranged from 1:100 to 1:25,600, and the IFA IgM titers ranged from 1:100 to 1:800. The overall prevalence of scrub typhus exposure was 48.0% (233 out of 485 participants). Visiting the rice field (82.0%) was detected as the most reported outdoor activity for seropositive participants, followed by visiting the corn field (79.0%) and firewood cutting (76.8%). Almost all seropositive participants reported wearing long sleeves and long pants (90.6%), wearing boots (89.7%), and wearing socks while working in the field (73.0%) (Table 2).

**Table 2:** Comparisons of outdoor activities and behaviors related to scrub typhus exposure by tribes

**Exposure**	**Total** n (%)	**Akha** n (%)	**Lahu** n (%)	**Hmong** n (%)	**Lisu** n (%)	**Yao** n (%)	**χ**^**2**^	***p*-value**
**Total**	**233(100.0)**	**125(53.7)**	**47(20.2)**	**15(6.4)**	**24(10.3)**	**22(9.4)**	**N/A**	**N/A**
**Sitting directly on the lawn without mats during work**								
Never	55(23.6)	26(20.8)	4(8.5)	6(40.0)	12(50.0)	7(31.8)	18.82	0.001*
Ever	78(76.4)	99(79.2)	43(91.5)	9(60.0)	12(50.0)	15(68.2)		
**Wild mushroom collecting**								
Never	87(37.3)	48(38.4)	12(25.5)	7(46.7)	10(41.7)	10(45.5)	4.23	0.376
Ever	146(62.7)	77(61.6)	35(74.5)	8(53.3)	14(58.3)	12(54.5)		
**Bamboo shoot collecting**								
Never	54(23.2)	33(26.4)	9(19.1)	1(6.7)	7(29.2)	4(18.2)	4.25	0.374
Ever	179(76.8)	92(73.6)	38(80.9)	14(93.3)	17(70.8)	18(81.8)		
**Firewood cutting**								
Never	50(21.5)	23(18.4)	12(25.5)	3(20.0)	7(29.2)	5(22.7)	2.33	0.684^a^
Ever	183(78.5)	102(81.6)	35(74.5)	12(80.0)	17(70.8)	17(77.3)		
**Bamboo cutting for basketry**								
Never	143(61.4)	71(56.8)	28(59.6)	13(86.7)	16(66.7)	15(68.2)	5.93	0.205
Ever	90(38.6)	54(43.2)	19(40.4)	2(13.3)	8(33.3)	7(31.8)		
**Straw for making brooms collecting**								
Never	134(57.5)	74(59.2)	25(53.2)	6(40.0)	13(54.2)	16(72.7)	4.58	0.333
Ever	99(42.5)	51(40.8)	22(46.8)	9(60.0)	11(45.8)	6(27.3)		
**Grass cutting**								
Never	134(57.5)	61(48.8)	30(63.8)	14(93.3)	16(66.7)	13(59.1)	13.37	0.010*
Ever	99(42.5)	64(51.2)	17(36.2)	1(6.7)	8(33.3)	9(40.9)		
**Bringing straw for feeding cows and buffaloes**								
Never	203(87.1)	133(90.4)	41(87.2)	15(100.0)	20(83.3)	14(63.6)	11.95	0.011*^, a^
Ever	30(12.9)	12(9.6)	6(12.8)	0(0.0)	4(16.7)	8(36.4)		
**Visiting the rice field**								
No	42(18.0)	32(25.6)	7(14.9)	1(6.7)	1(4.2)	1(4.5)	11.50	0.017*^, a^
Yes	191(82.0)	93(74.4)	40(85.1)	14(93.3)	23(95.8)	21(95.5)		
**Visiting the corn field**								
No	49(21.0)	32(25.6)	10(21.3)	3(20.0)	1(4.2)	3(13.6)	6.61	0.148^a^
Yes	184(79.0)	93(74.4)	37(78.7)	12(80.0)	23(95.8)	19(86.4)		
**Visiting the pineapple plantation**								
No	215(92.3)	119(95.2)	42(89.4)	14(93.3)	24(100.0)	16(72.7)	12.23	0.007*^, a^
Yes	18(7.7)	6(4.8)	5(10.6)	1(6.7)	0(0.0)	6(27.3)		
**Visiting the banana orchard**								
No	154(66.1)	83(64.4)	29(61.7)	12(80.0)	12(50.0)	18(81.8)	6.91	0.141
Yes	79(33.9)	42(33.6)	18(38.3)	3(20.0)	12(50.0)	4(18.2)		
**Visiting the tea plantation**								
No	68(29.2)	10(8.0)	20(42.6)	13(86.7)	4(16.7)	21(95.5)	103.76	< 0.001*^, a^
Yes	165(70.8)	155(92.0)	27(57.4)	2(13.3)	20(83.3)	1(4.5)		
**Visiting the coffee plantation**								
No	160(68.7)	76(60.8)	41(87.2)	14(93.3)	9(37.5)	20(90.9)	31.26	< 0.001*^, a^
Yes	73(31.3)	49(39.2)	6(12.8)	1(6.7)	15(62.5)	2(9.1)		
**Wearing long sleeves**								
Never	8(3.4)	2(1.6)	1(2.1)	0(0.0)	3(12.5)	2(9.1)	10.48	0.126^a^
Sometimes	14(6.0)	8(6.4)	4(8.5)	0(0.0)	2(8.3)	0(0.0)		
Everyday	211(90.6)	155(92.0)	42(89.4)	15(100.0)	19(79.2)	20(90.9)		
**Wearing long pants**								
Never	6(2.6)	2(1.6)	2(4.3)	0(0.0)	1(4.2)	1(4.5)	11.04	0.103^a^
Sometimes	16(6.9)	5(4.0)	6(12.8)	0(0.0)	4(16.6)	1(4.5)		
Everyday	211(90.6)	188(94.4)	39(83.0)	15(100.0)	19(79.2)	20(91.0)		
**Wearing boots**								
Never	9(3.9)	3(2.4)	4(8.5)	0(0.0)	1(4.2)	1(4.5)	7.84	0.325^a^
Sometimes	15(6.4)	11(8.8)	4(8.5)	0(0.0)	0(0.0)	0(0.0)		
Everyday	209(89.7)	111(88.8)	39(83.0)	15(100.0)	23(65.8)	21(95.5)		
**Wearing socks**								
Never	41(17.6)	14(11.2)	20(42.6)	0(0.0)	4(16.7)	3(13.6)	28.74	0.001*^, a^
Sometimes	22(9.4)	17(13.6)	3(6.4)	0(0.0)	0(0.0)	2(9.1)		
Everyday	170(73.0)	94(75.2)	24(51.0)	15(100.0)	20(83.3)	17(77.3)		
**Wearing gloves**								
Never	82(35.2)	32(25.6)	27(57.4)	6(40.0)	10(41.7)	7(31.8)	20.03	0.010*^, a^
Sometimes	69(29.6)	43(34.4)	9(19.1)	4(26.7)	9(37.5)	4(18.2)		
Everyday	82(35.2)	50(40.0)	11(23.4)	5(33.3)	5(20.8)	11(53.3)		
**Washing clothes immediately after work**								
Never	27(11.6)	12(9.6)	11(23.4)	0(0.0)	3(12.5)	1(4.5)	24.20	0.001*^, a^
Sometimes	71(30.5)	36(28.8)	19(40.4)	0(0.0)	7(29.2)	9(40.9)		
Everyday	135(57.9)	77(61.6)	17(36.2)	15(100.0)	14(58.3)	12(54.5)		

^*^Statistically significant at α = 0.05, ^a^Fisher exact test

Regarding outdoor activities, the Akha seropositive participants had the highest rates of visiting tea plantations (92.0%), cutting grass (51.2%), and cutting bamboo for basketry (46.4%). Almost all Lahu seropositive participants sat directly on the lawn without mats during work (91.5%), and 74.5% had collected mushrooms. Hmong seropositive participants had the highest rate of collecting bamboo shoots (93.3%) and straw for making brooms (60.0%). A total of 95.8% of the Lisu seropositive participants had the highest rate of visiting rice and corn fields, 62.5% visited coffee plantations, and 50.0% visited banana plantations. While 36.4% of the Yao seropositive participants had a history of bringing straw to feed cows and buffaloes, 37.3% visited the pineapple plantation and 53.3% wore gloves, which was higher than the proportions in other tribes (Table 2).

Ten (10) activities that showed statistical significance among seropositive participants from different tribes: sitting directly on the lawn without mats during work (p value = 0.001), cutting grass (*p* value = 0.010), bringing straw to feed cows and buffaloes (*p* value = 0.011), visiting rice fields (*p* value = 0.017), visiting pineapple plantations (*p* value = 0.007), visiting tea plantations (*p* value < 0.001), visiting coffee plantations (p value < 0.001), wearing socks (*p* value = 0.001), wearing gloves (*p* value = 0.010), and washing clothes immediately after work (*p* value = 0.011) (Table 2).

In the univariate model, 10 variables were found to be associated with scrub typhus exposure: age, tribe, marital status, education level, sitting directly on the lawn without mats during work, cutting grass, visiting tea or coffee plantations, wearing gloves, having other animals at home, having to contact with an animal at home (Table 3).

**Table 3:** Univariate and multivariate analyses in identification factors associated with scrub typhus exposure

Factors	Scrub typhus exposure		OR	95% CI	*p*-value	AOR	95% CI	*p*-value
	Yes n (%)	No n (%)						
**Total**	**233 (48.0)**	**252 (52.0)**	**N/A**	**N/A**	**N/A**	**N/A**	**N/A**	**N/A**
**Sex**								
Male	110(52.9)	98(47.1)	1.41	0.98–2.02	0.065			
Female	123(44.4)	154(55.6)	1.00					
**Age** (years)								
18–29	10(18.9)	43(81.1)	1.00			1.00		
30–39	28(31.5)	61(68.5)	1.97	0.87–4.49	0.104	1.91	0.80–4.53	0.143
40–49	41(45.6)	49(54.4)	3.60	1.61–8.04	0.002*	2.23	0.92–5.41	0.078
50–59	60(55.6)	48(44.4)	5.38	2.45–11.79	< 0.001*	3.47	1.41–8.55	0.007*
≥ 60	94(64.8)	51(35.2)	7.93	3.68–17.08	< 0.001*	4.31	1.73–10.72	0.002*
**Tribe**								
Akha	125(55.6)	100(44.4)	2.02	1.29–3.17	0.002*	2.20	1.31–3.72	0.003*
Lisu	24(53.3)	21(46.7)	1.85	0.93–3.68	0.081	1.49	0.68–3.23	0.318
Hmong	15(35.7)	27(64.3)	0.90	0.43–1.86	0.773	1.08	0.48–2.46	0.851
Yao	22(44.0)	28(56.0)	1.27	0.65–2.47	0.481	0.89	0.42–1.87	0.755
Lahu	47(38.2)	76(61.8)	1.00			1.00		
**Marital status**								
Single	13(26.0)	37(74.0)	1.00					
Married	173(48.7)	182(51.3)	2.71	1.39–5.26	0.003*			
Ever married	47(58.8)	33(41.2)	4.05	1.87–8.78	< 0.001*			
**Religion**								
Buddhist	111(51.9)	103(48.1)	1.32	0.92–1.89	0.134			
Christian	122(45.0)	149(55.0)	1.00					
**Education**								
Illiterate	206(57.2)	154(42.8)	4.86	3.02–7.80	< 0.001*	3.46	1.93–6.21	< 0.001*
Primary school and above	27(21.6)	98(78.4)	1.00			1.00		
**Having Thai ID card**								
No	42(41.6)	59(58.4)	0.72	0.46–1.12	0.145			
Yes	191(49.7)	193(50.3)	1.00					
**Having free access to health care service**								
No	29(37.7)	48(62.3)	1.00					
Yes	176(49.7)	178(50.3)	1.64	0.99–2.71	0.056			
Others	28(51.9)	26(48.1)	1.78	0.88–3.61	0.108			
**Annual household income** (bath)								
≤ 30,000	94(46.8)	107(53.2)	1.15	0.53–2.49	0.725			
30,001–50,000	65(43.3)	85(56.7)	1.00	0.45–2.21	1.000			
50,001–100,000	61(58.7)	43(41.3)	1.86	0.82–4.22	0.140			
≥ 100,001	13(43.3)	17(56.7)	1.00					
**Knowledge regarding scrub typhus prevention and control**								
Low	130(50.8)	126(49.2)	1.16	0.71–1.88	0.556			
Moderate	62(43.7)	80(56.3)	0.87	0.51–1.49	0.609			
High	41(47.1)	46(52.9)	1.00					
**Sitting directly on the lawn without mats during work**								
Never	55(48.2)	59(51.8)	1.00					
Sometimes	50(33.1)	101(66.9)	0.53	0.32–0.88	0.013*			
Often	128(58.2)	92(41.8)	1.49	0.95–2.35	0.084			
**Grass cutting**								
Never	134(43.5)	174(56.5)	1.00			1.00		
Sometimes	94(56.0)	74(44.0)	1.65	1.13–2.41	0.010*	1.85	1.20–2.84	0.005*
Often	5(55.6)	4(44.4)	1.62	0.43–6.16	0.477	1.21	0.29–5.02	0.792
**Visiting tea or coffee plantation**								
No	89(42.6)	120(57.4)	1.00					
Yes	144(52.2)	132(47.8)	1.47	1.02–2.11	0.037*			
**Wearing long sleeves**								
Never	8(57.1)	6(42.9)	1.00					
Sometimes	14(30.4)	32(69.6)	0.33	0.10–1.12	0.076			
Everyday	211(49.6)	214(50.4)	0.74	0.25–2.17	0.582			
**Wearing glove**								
Never	82(59.0)	57(41.0)	2.00	1.29–3.11	0.002*	2.12	1.28–3.49	0.003*
Sometimes	69(46.0)	81(54.0)	1.18	0.77–1.82	0.432	1.52	0.93–2.46	0.092
Everyday	82(41.8)	114(58.2)	1.00			1.00		
**Having other animals live at or near home**								
No	112(43.6)	145(56.4)	1.00					
Yes	121(53.1)	107(46.9)	1.46	1.02–2.09	0.037*			
**Having contacting with animal at home**								
Never	75(54.7)	62(45.3)	1.09	0.69–1.69	0.710			
Sometimes	59(36.9)	101(63.1)	0.53	0.34–0.81	0.003*			
Everyday	99(52.7)	89(47.3)	1.00					

^*^Statistically significant at α = 0.05

Five (5) variables were found to be associated with scrub typhus exposure in multivariate analysis. People aged 60 years and over and 50–59 years had a 4.31-fold (95% CI = 1.73–10.72) and 3.47-fold (95% CI = 1.41–8.55) increased risk of scrub typhus exposure than those aged less than 30 years, respectively. Those who were illiterate had a 3.46-fold (95% CI = 1.93–6.21) increased risk of scrub typhus exposure compared to those who had a primary school education and above. Akha people had a 2.20-fold (95% CI = 1.31–3.72) increased risk of scrub typhus exposure compared to Lahu people. Those who had a history of cutting grass had a 1.85-fold (95% CI = 1.20–2.84) increased risk of scrub typhus exposure compared to those who did not. Those who never wore gloves had a 2.12-fold (95% CI = 1.28–3.49) increased risk of scrub typhus exposure than those who wore gloves every day (Table 3).

## Discussion

The hill tribe people in northern Thailand had limited education and a low economic status. A large proportion of the hill tribe people had poor knowledge about scrub typhus prevention and control. They had a high risk of being exposed to scrub typhus due to their living environments and exposures, with most participants visiting several types of fields or plantations. The vast majority of participants reported using clothes that covered most of their body, including boots and socks, with only one-fourth not doing so. The important exception was gloves, which were only regularly worn by 40.4% of the participants. Almost half of the hill tribe people living in Chiang Rai, Thailand, were infected by scrub typhus (48.0%). Additionally, age, education level, tribe, cutting grass, and never wearing gloves were determined to be potential risk factors for scrub typhus exposure.

A large proportion (48.0%) of the hill tribe people living in Thailand who were aged 18 years old and over were ever infected by scrub typhus. Several studies conducted in countries in Tsutsugamushi Triangle areas also reported prevalence rates of 10.0% in China [29], 35.2% in Korea [30], and 34.7–40.0% in India [31, 32]. Interestingly, studies from Laos and Myanmar reported lower prevalence than that among the hill tribes in Thailand, at 20.3% and 19.0% [33, 34], respectively. Several studies conducted in other regions of Thailand reported a lower prevalence than that among hill tribe people, with rates of 31.8% in the central region [35] and 4.2% [36] in the northeastern region of Thailand. The particularly high prevalence in the study population reflects the generally high incidence in northern Thailand and a potentially higher risk in the population of hill tribe members, who perform many high-risk activities. Many outdoor activities performed related the scrub typhus exposure among the tribes were found to be statistically different proportions such as sitting directly on the lawn without mats during work experience, grass-cutting experience, visiting the rice field, visiting the pineapple plantation, visiting coffee and tea plantations, including wearing gloves in their daily life. These activities reflected their socioeconomic status which was associated with the scrub typhus exposure.

Being older age was detected as one of the potential risk factors for scrub typhus exposure among hill tribe people in Thailand. This coincides with studies in India [37–39] and Myanmar [34], which reported that the risk of exposure was strongly associated with increasing age. Studies conducted in Japan and South Korea confirmed that older participants were more susceptible to scrub typhus infection than younger participants [10]. However, a case‒control study conducted in China did not report such a link [40]. One possible explanation could be that older people have a higher possibility to exposure repeatedly to the organism over a longer period of time, then thy have higher levels of and longer-persisting antibodies eventually.

In our study, those who never attended school were at a greater risk of scrub typhus exposure. Schooling provides the opportunity to attain language proficiency and the subsequent understanding of health information. Similar findings were reported in India [37, 38], Laos [33] and the northeast and southern regions of Thailand [35], with low education levels being associated with greater risks of scrub typhus infection. In contrast, several studies conducted in Vietnam [41], Korea [42], and China [40] did not show any link between education level and scrub typhus infection. This might be because almost all participants had the same educational level, and thus, there may not be enough statistical power to detect differences. Considering that hill tribe occupations mainly involve fieldwork, it is plausible that the risk factors for scrub typhus are more closely associated with occupational features than with individuals' education levels.

Our study explored the links between knowledge, income, illiteracy, and the presence of scrub typhus antibodies among hill tribe people in northern Thailand. While we found a connection between illiteracy and scrub typhus antibodies, there was no significant association between the presence of scrub typhus antibodies and knowledge or income. This indicates a more complex relationship among these variables. To better understand scrub typhus exposure in this population, further investigations and a comprehensive analysis are essential. This should encompass an examination of cultural practices and beliefs alongside education and income.

The risk of scrub typhus exposure appeared to vary among the different tribes in our study. The Akha tribe exhibited the highest prevalence (55.6%), followed by the Lisu (53.3%) and Yao tribes (44.0%). A previous study by Wangrangsimakul et al. [43] also reported that the Akha and Lahu tribes were at greater risk of scrub typhus infection than other tribes. This may be secondary to dressing style while working in farms. Akha and Yao women [44] always wear miniskirts, while Lisu men wear loose trousers [44], which could make it easier for them to be infected than individuals in other tribes who wear tight-fitting clothing according to their culture and lifestyles. Moreover, hill tribe people in Thailand who did not use gloves had a greater risk of scrub typhus exposure. Several studies also reported that wearing gloves could protect individuals from scrub typhus infection [11, 45]. Other clothing has been reported to be protective against scrub typhus infection, including long sleeve garments, long pants, boots, and socks [11, 40, 41, 46].

The study revealed significant participant contact with both domestic animals like chickens and dogs at home and wild animals such as rodents, squirrels, and chipmunks at their workplace, all of which share the human environment. Similar findings in studies conducted in Malaysia and India have also highlighted an increased scrub typhus risk linked to close contact with domestic animals [12, 47, 48]. However, our univariate analysis demonstrated that those in close contact with other domestic animals like cats, pigs, or ducks at home faced a higher risk of scrub typhus exposure. This suggests that specific domestic animals may indeed raise the risk of the people exposure rate. Nonetheless, the final model did not establish a significant association between overall animal exposure and scrub typhus exposure. It's crucial to take note of this result and exercise caution when in contact with these animals, as there remains a potential infection risk.

Additionally, as chiggers have been reported in various ecological settings, including fields, agricultural land, mountainous regions, forests and forest edges, rice fields, grasslands, parks, riversides, orchards, and plantations [9]. This study identified environmental exposure to various plantations as potential risk factors for scrub typhus exposure, including rice fields, corn fields, tea plantations, and coffee plantations. Many participants who visited these plantations were detected the antibodies with scrub typhus. Additionally, participants living near trees, bushes, piles of leaves, or damp land had higher exposure rates. Other studies found similar risk factors, like water, rodents, dry field farming, and livestock exposure [9–11]. However, the final model did not confirm the association between plantations and scrub typhus. Nevertheless, caution is advised when visiting such areas due to potential disease transmission. Wearing protective equipment (PPE) is strongly recommended to prevent the exposure.

This study had a few limitations. First, as the nature of a cross-sectional study design, possible recall bias due to the inability to recall some events in the past were limitations of the study. Second, the cutoff point used in the study and the decline in IgG antibody levels two years after exposure [39], which is a common feature of many infectious diseases, might impact the estimation of the prevalence and the analysis of the rest of the study. Third, the period of data and blood sample collection was not conducted in the high infection season of the disease, so the whole year cycle of the infection might not have been presented. Most exposure occurs in summer, when people prepare their farms for planting.

## Conclusions

A large proportion of the hill tribes living in northern Thailand have been exposed scrub typhus from their daily lifestyles. Elderly, illiterate, and Akha people are at particularly high risk. Cutting grass is associated with scrub typhus exposure; people who never use protective clothing or gear are also at substantial risk of scrub typhus exposure. Taken together, campaigns to improve scrub typhus awareness and effective public health interventions need to be urgently developed. Promoting to wear proper suit while farming particularly cutting grass should be implemented in the hill tribe communities. Health education program regarding scrub typhus prevention and control should be also consideration particularly the elderly population and Akha people.

## Data Availability

The datasets used and/or analysed during the current study available from the corresponding author on reasonable request.

## Abbreviations

CI: Confident interval
IOC: Item-objective congruence
BMI: Body mass index
ELISA: Enzyme linked immunosorbent assay
IFA: Imunofluorescence assay
